# Microtubule dependent changes in cell polarity are required for mammary epithelial cell migration and branching morphogenesis

**DOI:** 10.1242/bio.062267

**Published:** 2025-12-11

**Authors:** Andrew K. Fraser, Joshua Carreras, Isabel A. Ryan, Jennifer Zoll, Andrew J. Ewald

**Affiliations:** ^1^ Department of Cell Biology, Giovanis Institute of Translational Cell Biology, and Center for Cell Dynamics, School of Medicine, Johns Hopkins University, Baltimore, MD 21205, USA; ^2^ Department of Biomedical Engineering, School of Medicine, Johns Hopkins University, Baltimore, MD 21205, USA

**Keywords:** Branching morphogenesis, Mammary epithelium, Microtubules

## Abstract

Branching morphogenesis requires dramatic changes in cell polarity, proliferation, migration, and actin dynamics to elaborate tubular networks. However, little is known about how microtubules support these cell behaviors in 3D tissues. Using organotypic cultures, we first examined the organization of the microtubule cytoskeleton. Simple luminal epithelial cells exhibited non-centrosomal, apico-basally oriented microtubule arrays, while stratified luminal cells had centrosomally radiating microtubules. During collective migration, luminal cells adopted an ameboid-like organization with a rear-facing nuclear-centrosomal axis. Multiple staining approaches suggest that cells in the basal-most luminal cell layer had more stable microtubules than cells deeper within the stratified layer. Finally, we tested the requirement for microtubules using pharmacologic inhibitors. Both microtubule stabilization and destabilization prevented bud formation and arrested duct elongation. Cell tracking analysis demonstrated that microtubules coordinated luminal cell migration within elongating buds. Destabilizing microtubules reduced cell directionality, while stabilizing microtubules did not affect directionality but reduced cell motility. Our data reveal that microtubules are essential for collective migration of luminal cells and for mammary branching morphogenesis.

## Teaser

**Summary:** Luminal epithelial cells utilize characteristic transitions in microtubule organization and dynamics to coordinate 3D collective migration and accomplish mammary branching morphogenesis.

## INTRODUCTION

At birth, female mice have a rudimentary mammary duct composed of bilayered epithelium (Hogg et al., 1983). Hormonal signals during puberty induce branching morphogenesis. The epithelium then branches out into the surrounding mesenchyme to form a mature ductal network filling the mammary fat pad (Hinck and Silberstein, 2005; Sternlicht, 2006). The rudiment and the mature duct share a bilayered architecture with quiescent, polarized luminal cells forming a simple, cuboidal epithelial tube surrounded by the myoepithelium and basement membrane. During puberty, luminal epithelial cells at the tips of ducts divide asymmetrically, resulting in stratified epithelium with robust proliferation, low polarity, and dynamic cell rearrangements (Ewald et al., 2008, 2012; Huebner et al., 2014). Cells lining the lumen show aspects of apical-basal polarity, but migratory interior cells have front-rear polarity. This stratified epithelium elongates through the collective migration of luminal cells (Huebner et al., 2016). Over time, radial intercalation and boundary capture of migratory luminal cells at the basal tissue surface resolves the stratified luminal epithelium to a polarized monolayer and expanded ductal tree (Neumann et al., 2018). The involvement of microtubules in polarity, proliferation and migration of cells in 2D culture suggests they could play a critical role in duct elongation, but this has yet to be explored.

Across organisms and tissues studied, polarized simple epithelial cells organize stable microtubules in apicobasally oriented non-centrosomal arrays (Muroyama and Lechler, 2017a,b). While centrioles are present near the apical surface, microtubules organize from the apical cortex via the minus-end binding protein CAMSAP3 (Toya et al., 2016). EpH4 cells, a mammary cell line, adopt this apicobasal arrangement in 2D culture but this has not, to our knowledge, yet been confirmed in 3D culture or *in vivo* (Yano et al., 2013). Several studies have identified roles for microtubules in shaping epithelial tissues including polarization (Muroyama et al., 2018), lumen formation (Akhtar and Streuli, 2013), keratinization (Muroyama and Lechler, 2017b), apical constriction (Ko et al., 2019), and mechanical homeostasis (Mitsuhata et al., 2021). Comparatively less is known about the organization and function of microtubules in epithelial cells during collective migration and branching morphogenesis. Dynamic, centrosomal microtubules are commonly required for mesenchymal and amoeboid single-cell migration in 3D (Bouchet and Akhmanova, 2017) as well as some forms of epithelial migration, including angiogenesis (Martin et al., 2018), wound healing (Wu et al., 2011), and metastasis (Wu et al., 2011). In *in vitro* models of EMT, epithelial cells switch to centrosomal arrays before disseminating (Hernandez and Tirnauer, 2010). It has been proposed that a similar switch occurs in epithelial buds during mammary development, but this has not yet been demonstrated directly.

Microtubules are critical for establishing cell polarity and coordinating an intracellular organization consistent with that polarity. The polarity axis is defined by membrane protein localization and actin organization, while intracellular polarization affects centrosomal and organelle positioning. Polarity is often assessed using the nuclear-centrosomal axis, which describes the relative positions of the nucleus and centrosome (Luxton and Gundersen, 2011), with the Golgi commonly located near the centrosome. In differentiated simple epithelium, non-centrosomal microtubule arrays maintain the basal position of the nucleus and apical position of the centrosome and Golgi (Toya et al., 2016). Lumen formation in 3D cultured mammary acini requires an apically oriented nuclear-centrosomal axis (Akhtar and Streuli, 2013). In migratory cells, microtubules maintain an intracellular organization consistent with their front-rear polarity and mode of migration. The centrosomal microtubule organizing center (MTOC) and Golgi organize in front of the nucleus during mesenchymal migration but behind the nucleus during ameboid migration (Yamada and Sixt, 2019). In tissue sections of mammary endbuds, Golgi are occasionally observed near the basal surface at the migration front. This configuration has led to the inference that luminal cells undergo partial-EMT at the onset of branching, inverting the nuclear-centrosomal axis and adopting mesenchymal-like motility (Burute et al., 2017). However, centrosomal MTOC position and Golgi dynamics of mammary epithelial cells migrating within 3D tissues have not been confirmed.

We set out first to characterize microtubule organization in the mammary gland and then test the role that microtubules play in branching morphogenesis. The sensitive dynamics and nano-scale structure of microtubules present significant challenges to *in vivo* studies, especially within internal organs in mammals. To overcome these barriers, we utilized 3D organotypic mammary culture. Primary mammary organoids maintain the heterogeneity, organization, and behaviors of native mammary tissue (Ewald et al., 2008, 2012; Huebner et al., 2014). These cultures are also well suited for timelapse imaging and for experimental perturbation of microtubules.

## RESULTS

### Mammary epithelial buds display microtubule reorganization at the elongating front

**Fig. 1.**
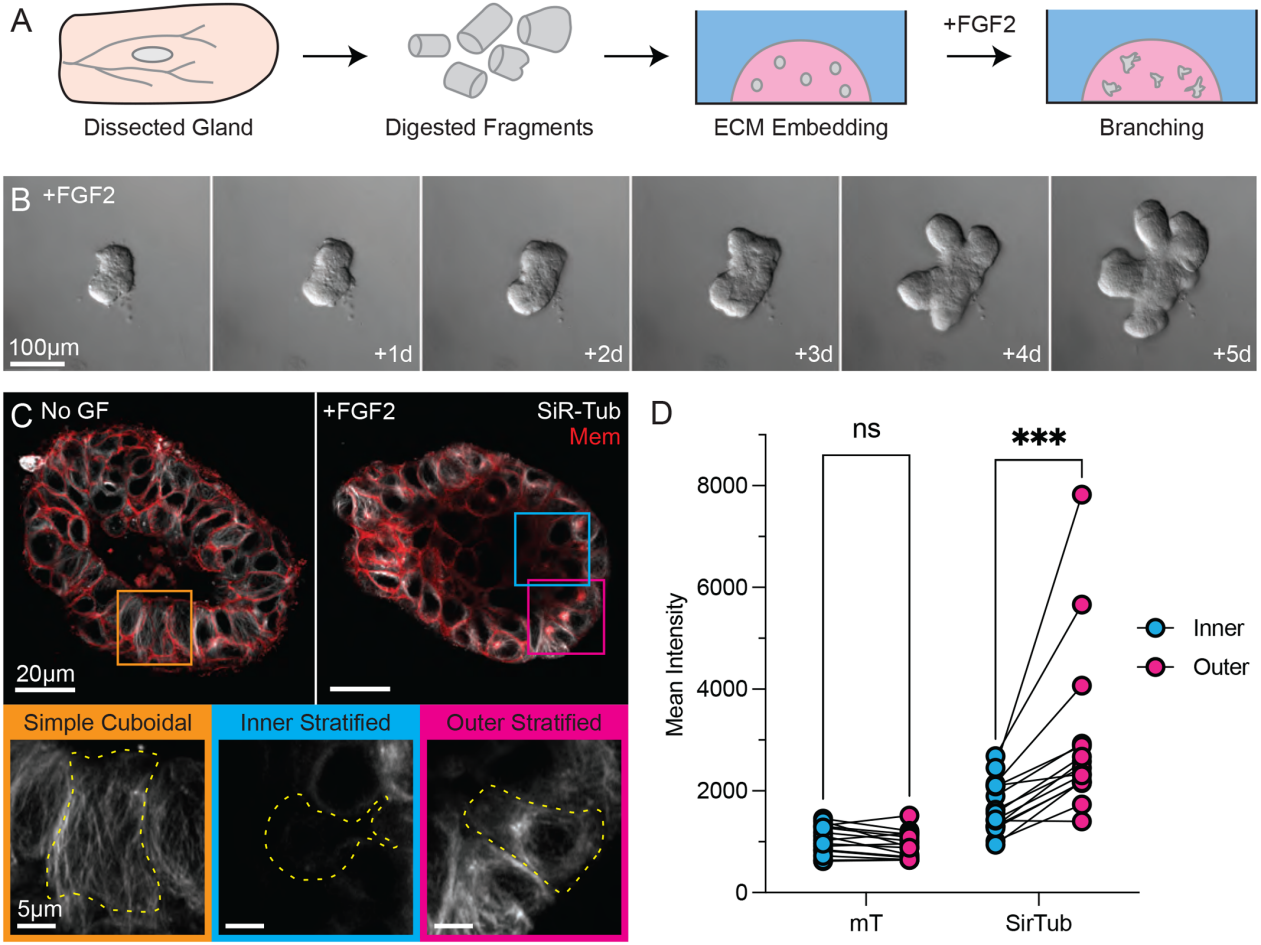
**Luminal cells reorganize their microtubule cytoskeleton during branching morphogenesis.** (A) Mammary organoid culture workflow. (B) DIC timelapse imaging of a representative organoid undergoing branching morphogenesis, following addition of FGF2. (C,D) Super-resolution images of mammary cysts (No GF) and buds (+FGF2). Insets show examples of luminal cells in simple cuboidal epithelium (orange) and stratified epithelial buds (cyan, inner; magenta, outer). (C) High magnification live imaging of microtubule organization of an elongating bud from a branching organoid (+FGF2) and a region of polarized epithelium from a control (NoGF) organoid, using vital dye (white=50 nM SiR-Tubulin, red=mTomato). (D) Quantification comparing fluorescence intensity of SirTubulin and mTomato (mT) between inner stratified and outer stratified cells of elongating buds (*n*=6 TEB regions from two biological replicates). ns, not significant; ***P<0.0001.

Previous work showed stable microtubules in polarized mammary acini in 3D culture and suggested an apical non-centrosomal MTOC (ncMTOC) organization (Peters et al., 2013; Xu et al., 2019; Yano et al., 2013). We hypothesized that since both the actin cytoskeleton and cell polarity reorganize during stratification and branching, the microtubules would also reorganize. To examine microtubules in live organoids, we used SiR-Tubulin, a fluorogenic microtubule dye (Lukinavicius et al., 2014). We note that SiR-Tubulin labels microtubules in an age-dependent manner, with older microtubules labeled preferentially (David et al., 2019). Accordingly, SiR-Tubulin has the advantage that it directly assesses the location of older, more stable microtubules and the disadvantage that it leaves the most dynamic microtubules unlabeled. Organoids were prepared from transgenic mice expressing fluorescent centriole (Centrin-GFP) and plasma membrane (membrane localized tdTomato) labels. Briefly, we enzymatically digest mammary glands, recover epithelial fragments by differential centrifugation, and suspend them in extracellular matrix (ECM; 1:1 Matrigel/Collagen I) (Fig. 1A,B) (Nguyen-Ngoc and Ewald, 2013; Nguyen-Ngoc et al., 2015). Organoids were cultured for 4-6 days, until active branching was evident, then processed for live or fixed imaging.

**Fig. 2.**
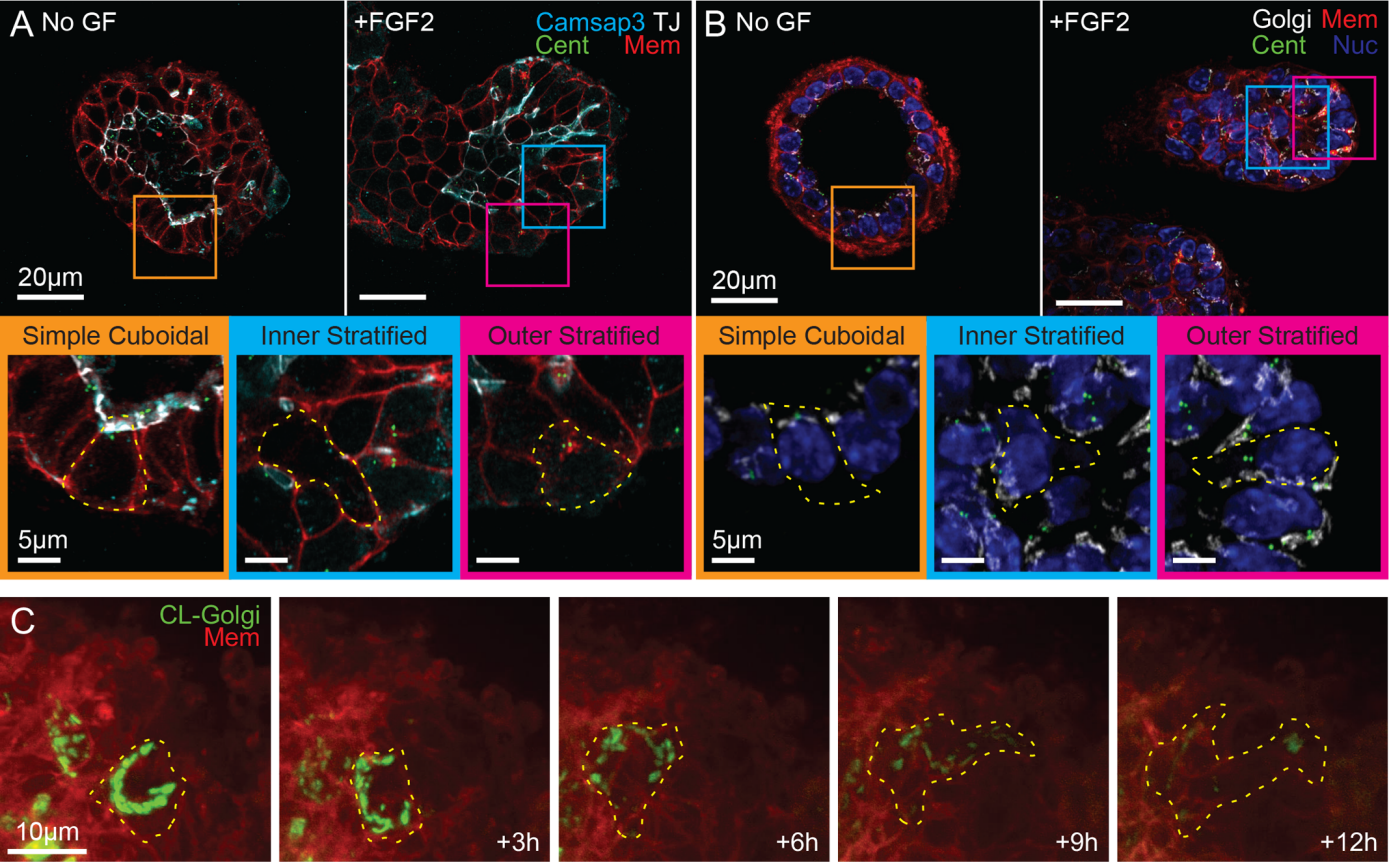
**Correlated changes in MTOC, Golgi, and associated microtubule in luminal cells.** (A) Super-resolution IF images of mammary cysts (No GF) and buds (+FGF2) stained for centrosomal MTOC proteins (white, Zo1; red, mTomato; green, Centrin-GFP; cyan, Camsap3). Insets show examples of luminal cells in simple cuboidal epithelium (orange) and stratified epithelial buds (cyan, inner; magenta, outer). (B) Super-resolution IF images of centriole and Golgi organization (white, GM130; red, mTomato; green, Centrin-GFP; blue, DAPI). (C) Representative spinning-disk confocal image sequence depicting Golgi reorientation during radial intercalation into the outer stratified layer (red, mTomato; green, CellLight™ Golgi).

We first examined organoids cultured in minimal media as a control. Under these conditions, organoids do not branch but instead form cysts with their luminal cells in a simple cuboidal arrangement surrounding a central lumen (Fig. 1C). SiR-Tubulin revealed microtubules arranged in apicobasal arrays (Fig. 1C). Centrioles (Centrin-GFP) were present near the apical membrane, but microtubules were not observed radiating from any focal point (Fig. 2A). Immunostaining of CAMSAP3, a well characterized minus end-binding protein known to localize to the apical cortex, revealed apical localization near the tight junctions in luminal cells (Fig. 2A). CAMSAP3 is therefore properly positioned for anchoring non-centrosomal apicobasally oriented microtubules.

We observed a notably different microtubule organization in branching epithelial buds of organoids treated with FGF2. Stratified luminal cells displayed radial arrays of microtubules emanating from a central node that coincided with the centriole (Figs 1C and 2B). CAMSAP3 was expressed only in cells contacting the luminal surface in the endbud, and it did not appear to serve as the MTOC (Fig. 2A). Apicobasal arrays were observed only in the simple cuboidal epithelium proximal to the bud. These observations suggest that while stratified luminal cells may specify an apical domain of CAMSAP3 localization, the apical ncMTOC is preferentially utilized in polarized, simple mammary epithelium.

### Microtubule reorganization correlates with a reorganized polarity axis

While differentiated mammary luminal cells had previously been shown to localize their centrioles and Golgi apically and to have apicobasally oriented microtubules (Xu et al., 2019), the MTOC and organelle localization utilized by migrating epithelial cells in 3D tissues is unknown. However, instances of Golgi localization near the basal surface of endbuds has led to the inference of a forward facing MTOC and mesenchymal-like migration (Burute et al., 2017). To examine intracellular polarization during branching morphogenesis directly, we performed immunofluorescence for Golgi (anti-GM130) in cultured organoids. In simple luminal epithelium, centrioles and Golgi oriented on the apical aspect of the nucleus, as expected (Fig. 2B). In elongating buds, nuclei are found at the rear of luminal cells. While centrosome and Golgi position was less stereotyped than in simple epithelium, they were typically restricted to the rear or lateral aspect of the nucleus (Fig. 2B). Inversion of the nuclear-centrosomal axis was not observed.

We could not rule out the possibility of transient nuclear-centrosomal axis inversion. We observed rare luminal cells with anti-GM130 staining in their leading protrusion as well as the Golgi and centriole staining at the rear of the cell (Fig. 2B). This led us to examine Golgi dynamics using time-lapse imaging. In organoids expressing CellLight™ Golgi-GFP, time-lapse imaging revealed examples of the Golgi reorienting from rear facing and perinuclear into basally directed cell protrusions (Fig. 2C). Maintaining a centrosomal MTOC at the rear in the ameboid style allows luminal cells to undergo transitions between apical-basal and front-rear polarity without inverting the intracellular polarization.

### Microtubules appear more dynamic in interior-located than basally located luminal cells

In elongating mammary endbuds, luminal cells within the interior of the stratified layer dynamically migrate and rearrange, but the outermost luminal cells adopt a columnar morphology and maintain their position in the boundary layer. Internal cells radially intercalate between outer cells and are captured into the boundary layer and cease protrusive migration (Neumann et al., 2018). One striking feature of microtubule imaging is the difference in microtubule signal between these inner and outer luminal cells (Fig. 1C). The basal-most layer of luminal cells showed microtubule density similar to that observed in simple luminal cells, while the interior-located luminal cells showed substantially diminished SiR-Tubulin signal (Fig. 1C). This difference was quantified across multiple elongating buds and revealed significantly higher SiR-Tubulin intensity in the outer, relative to inner, stratified luminal cells (Fig. S1A and Fig. 1D). No difference was observed in plasma membrane marker intensity between outer and inner cells (Fig. 1D). Time-lapse imaging of Sir-Tubulin showed no observable microtubule dynamics in the outer cells. These findings were confirmed in fixed samples by immunostaining with anti-α-tubulin antibody, with a strong IF signal in the outer layer of luminal cells that was nearly absent within the stratified layer (Fig. S1B). Similar results were obtained with a polyclonal pan-tubulin antibody, and it is known that our chosen fixation approach can preferentially preserve the most stable microtubules (Goldspink et al., 2017). These observations suggest that microtubules in the outer cell layers are more stable and therefore better preserved during fixation and better labeled by SiR-Tubulin.

### Luminal cell proliferation precedes bud extension

Our previous work demonstrated that mammary branching morphogenesis requires both proliferation and cell migration (Huebner et al., 2014, 2016). Proliferation stratifies the luminal epithelium and supplies additional cells for allocation into elongating ducts. Cell migration reorganizes those cells into a polarized and elongated tube. To better understand the role of cell proliferation in branching morphogenesis, we examined proliferative and cell cycle dynamics in the organoid assay. We cultured organoids in media supplemented with EdU for 24 h then dissociated the ECM to recover the organoids in suspension. Organoids were then trypsinized to single cells for flow cytometry. We collected samples daily for 8 days from three biological replicates to access proliferation by EdU incorporation and cell cycle by Propidium Iodide (PI) staining for DNA content. Proliferation rate peaked early on, with over 80% of cells positive for EdU during the second and third days of culture (Fig. S2A). From here proliferation declined significantly, falling below 25% of cells by day six. Similarly, cell cycle analysis revealed a peak of over 40% of cells in S or G2/M phase at 48 h before a steady decline with additional time in culture (Fig. S2B). From day five forward, the percentage of cells in interphase was above that measured at day zero before branching media was added. We conclude that FGF2 in the branching media stimulates a wave of epithelial cell proliferation that peaked during the first three days. This wave subsides as buds begin to form at culture day four and returns to homeostatic levels as epithelial buds begin to elongate into the surrounding ECM. Hence, consistent with previous data (Huebner et al., 2016), proliferation and cell-migration occur largely sequentially in mammary organoids. An early high-proliferation phase induces the luminal epithelium to stratify, followed by a low-proliferation phase during which luminal cell migration and intercalation extends the duct.

### Disrupting microtubule stability prevents epithelial bud formation

**Fig. 3.**
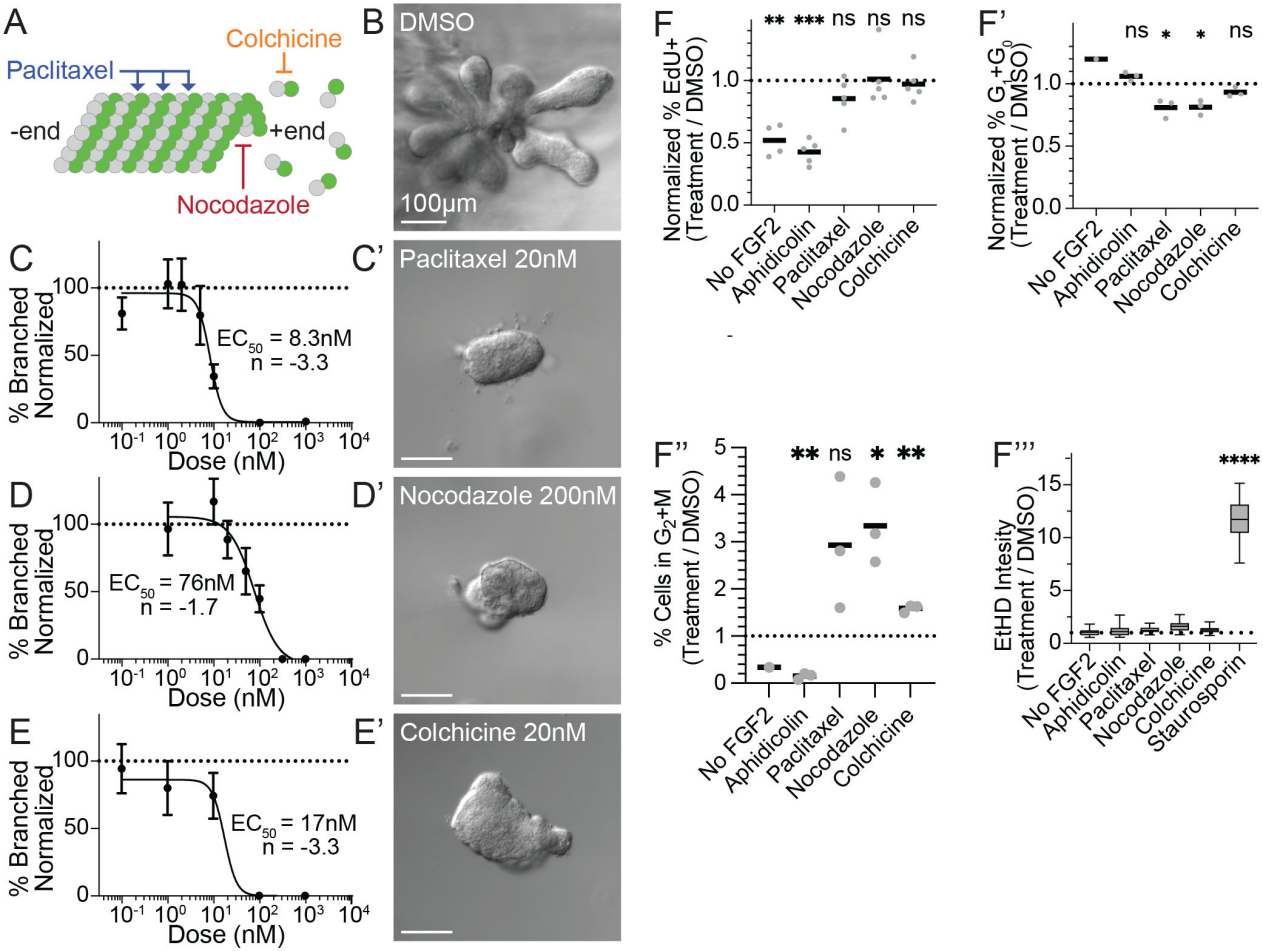
**Microtubule disruption prevents initiation of branching morphogenesis.** (A) Schematic representing activity of microtubule targeting agents paclitaxel (stabilizing), nocodazole (destabilizing), and colchicine (inhibits polymerization). (B) Representative DIC image of DMSO treated control organoid after 7 days of culture in FG2 media. (C-E) Branching morphogenesis dose-response curves for MTAs (r=5 mice). Branching accessed by the fraction of organoids with buds_treatment_ / fraction of organoids with buds_DMSO_. (C′-E′) DIC images of representative organoids from each condition after 7 days of culture. (F-F‴) Quantification of the normalized percentage of EdU incorporation (EdU, r=5), normalized percentage of interphase cells and percentage of cells in G2+M (PI, r=3), and cell death (mean EtHD intensity, r=5).

Since luminal cells reconfigure their microtubule cytoskeleton within the elongating bud, we hypothesized that these changes would be essential to branching morphogenesis. To test this hypothesis, we set out to perturb microtubules and observe any resulting defects in organoid morphology, using pharmacologic agents that alter microtubule stability. Paclitaxel stabilizes microtubules, nocodazole destabilizes microtubule plus ends, and colchicine binds free tubulin heterodimers (Fig. 3A). We note that we used nocodazole in the ‘low dose’ range; it was likely sufficient to inhibit microtubule plus-end dynamics but complete microtubule depolymerization typically requires 400 nM to 5 mM doses. We dosed organoids at the start of culture and then counted the percentage of organoids that bud by culture day seven. We tested a range of doses in three biological replicates to determine dose response curves. Paclitaxel (IC50=8.3 nM, h=−3.3; Fig. 3C), nocodazole (IC50=76 nM, h=−1.7; Fig. 3D), and colchicine (IC50=17 nM, h=−3.3, Fig. 3E) all prevented bud formation. The microtubule destabilizing agents vincristine and demecolcine yielded similar results (Fig. S2C-C′, D-D′). The branching response to nocodazole and paclitaxel is consistent with findings of others (Xu et al., 2019).

To understand the requirement for microtubules more deeply, we examined organoids treated with doses of each drug that reduce branching below 20% of control levels. At day seven, organoids branched vigorously in the vehicle treated control (Fig. 3B). All three drug treatments prevented budding (Fig. 3C′,D′,E′) although the organoids appeared viable and notably larger than organoids in minimal media. Because microtubule disruption can cause cell cycle arrest, we examined drug effects on proliferation, mitosis, and toxicity. As an additional positive control, we treated organoids with the antiproliferative agent aphidicolin, a DNA polymerization inhibitor that blocks mitotic entry, testing a range of doses in three biological replicates to determine a dose response curve. Consistent with our previous findings, blocking proliferation with aphidicolin prevented bud initiation at doses above 500 nM (IC50=150 nM, h=−1.6; Fig. S2E-E′). Twenty-four hours after treatment, only aphidicolin significantly reduced EdU incorporation (Fig. 3F), paclitaxel and nocodazole moderately reduced interphase cell % (Fig. 3F′), and nocodazole significantly increased the percentage of cells in G2+M (Fig. 3F″). Increased cell death was only observed in the apoptosis-inducing positive control, staurosporin (Fig. 3F″′). These results demonstrate that at doses sufficient to prevent bud formation, microtubule agents did not cause detectable toxicity or dramatic mitotic disruption. These results indicate that paclitaxel, nocodazole, and colchicine prevent bud formation to a degree not readily explainable by their apparent antimitotic effects, at these low doses. We concluded that epithelial bud initiation requires spatiotemporally regulated microtubule dynamics within the luminal epithelial cells.

### Disrupting microtubule stability prevents epithelial bud elongation

Given the importance of microtubules in bud initiation, we hypothesized they would be similarly critical to bud elongation. To test this hypothesis we again utilized paclitaxel, nocodazole, and colchicine but waited until culture day four to add the inhibitors. This allowed the early proliferative stage to complete and buds to initiate before disrupting microtubules, isolating the effects to actively elongating buds. Previous results show that blocking proliferation by treatment with aphidicolin at this timepoint had no acute effect on organoid branching (Huebner et al., 2016). After 72 h, we measured the projected area and number of branches for each organoid, testing a series of concentrations in three biological replicates to determine the dose response. All three microtubule perturbing drugs potently inhibited branching (Fig. S3A-C) and bud elongation (Fig. S3A′-C′), at the doses previously determined to prevent bud initiation. These doses were selected for further investigation by time-lapse microscopy.

**Fig. 4.**
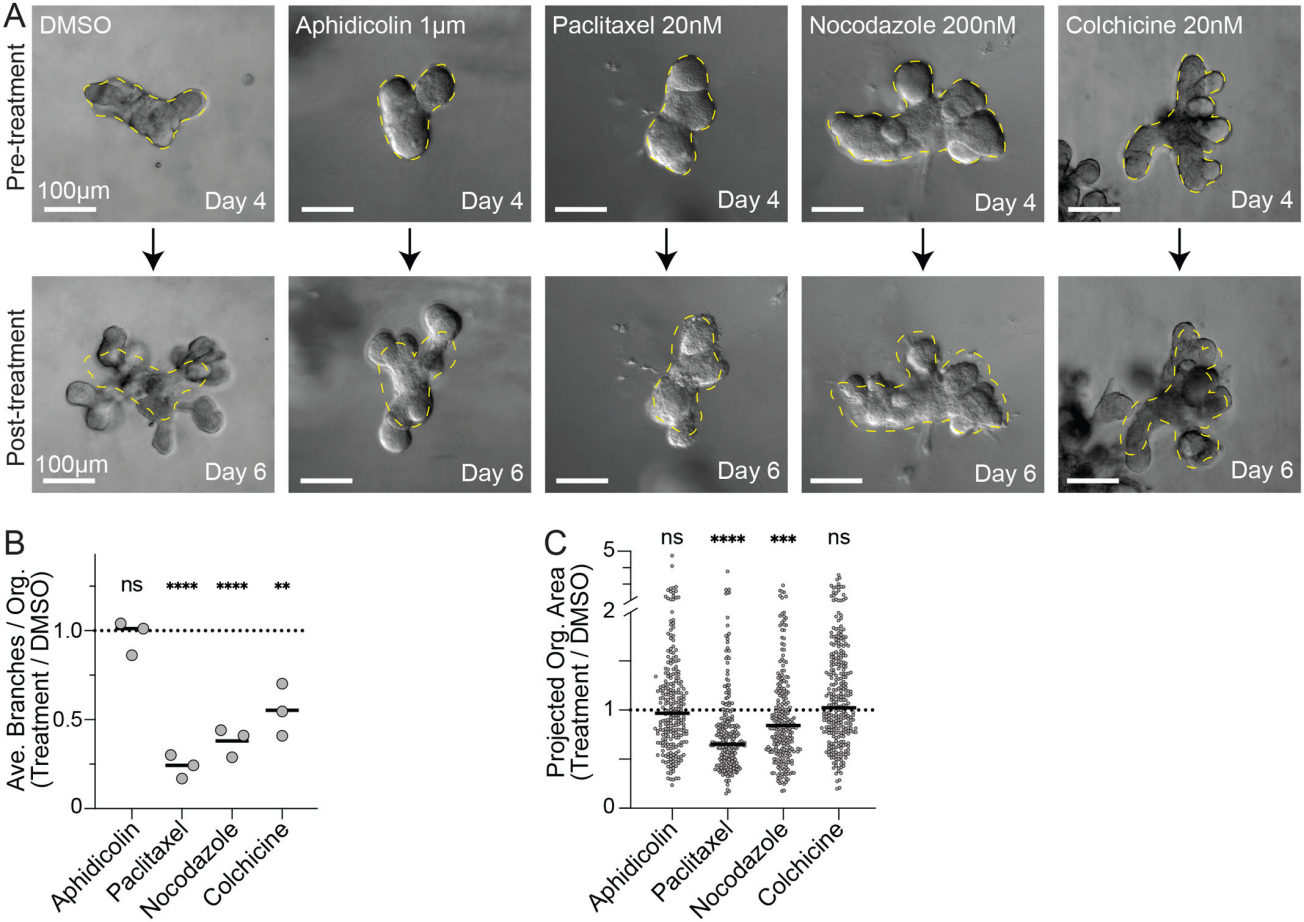
**Microtubule disruption prevents bud elongation and branching.** (A) Representative DIC images (A) before (day four) and after (day six) inhibitor addition. Branching (B) and growth (C) response for selected doses of MTAs.

In contrast to the continued branching observed in DMSO and aphidicolin controls, treatment with paclitaxel, nocodazole, or colchicine acutely arrested branching. After 48 h, existing buds failed to elongate or bifurcate in drug treated conditions (Fig. 4A). Examining several hundred organoids from three biological replicates confirmed these observations. Microtubule perturbation significantly decreased branch number in all three cases (Fig. 4B). Paclitaxel and nocodazole treatments also significantly decreased organoid size, while colchicine treated organoids were not significantly smaller than controls (Fig. 4C). Only aphidicolin had a significant effect on EdU incorporation (Fig. S3D). Only paclitaxel significantly altered the fraction of interphase cells (Fig. S3E), none of the three microtubule agents significantly increased the percentage of cells in G2+M (Fig. S3F), and only staurosporin treatment significantly increased cell death (Fig. S3G). These results demonstrated that microtubule disruption arrests bud elongation and branching despite having only modest mitotic effects, at these low doses.

### Actin dynamics determine the mode of luminal cell migration

The involvement of actin in branching morphogenesis is comparatively well established. Previous work in our lab showed that the actin arresting inhibitor cocktail JLY (jasplakinolide, latrunculin, Y27632) ablates luminal cell protrusions, halting cell migration and bud elongation (Neumann et al., 2018). However, the specific consequences of individually increasing or decreasing F-actin stability have not been characterized. We therefore examined the effects of treatment with the F-actin stabilizer jasplakinolide, the F-actin destabilizer cytochalasin D, and the G-actin toxin latrunculin D (Fig. S4A). At low doses, jasplakinolide decreased organoid branching, but doses above 20 nM induced protrusive invasion (Fig. S4C-C′), instead of the budding pattern observed in controls (Fig. S4B). This protrusive invasion resembled the behavior of organoids cultured in a stiff, fibrillar collagen I matrix (Nguyen-Ngoc and Ewald, 2013). Cytochalasin D (0.5 µM) prevented budding and caused cells to extend spindly protrusions into the ECM instead (Fig. S4D-D′). Latrunculin reduced branching over a broad therapeutic window but did not induce an invasive morphology (Fig. S4E-E′). These results reveal that disruption of the actin versus microtubule cytoskeleton has qualitatively different consequences for branching morphogenesis.

### Microtubules regulate luminal cell speed and directionality during collective migration

**Fig. 5.**
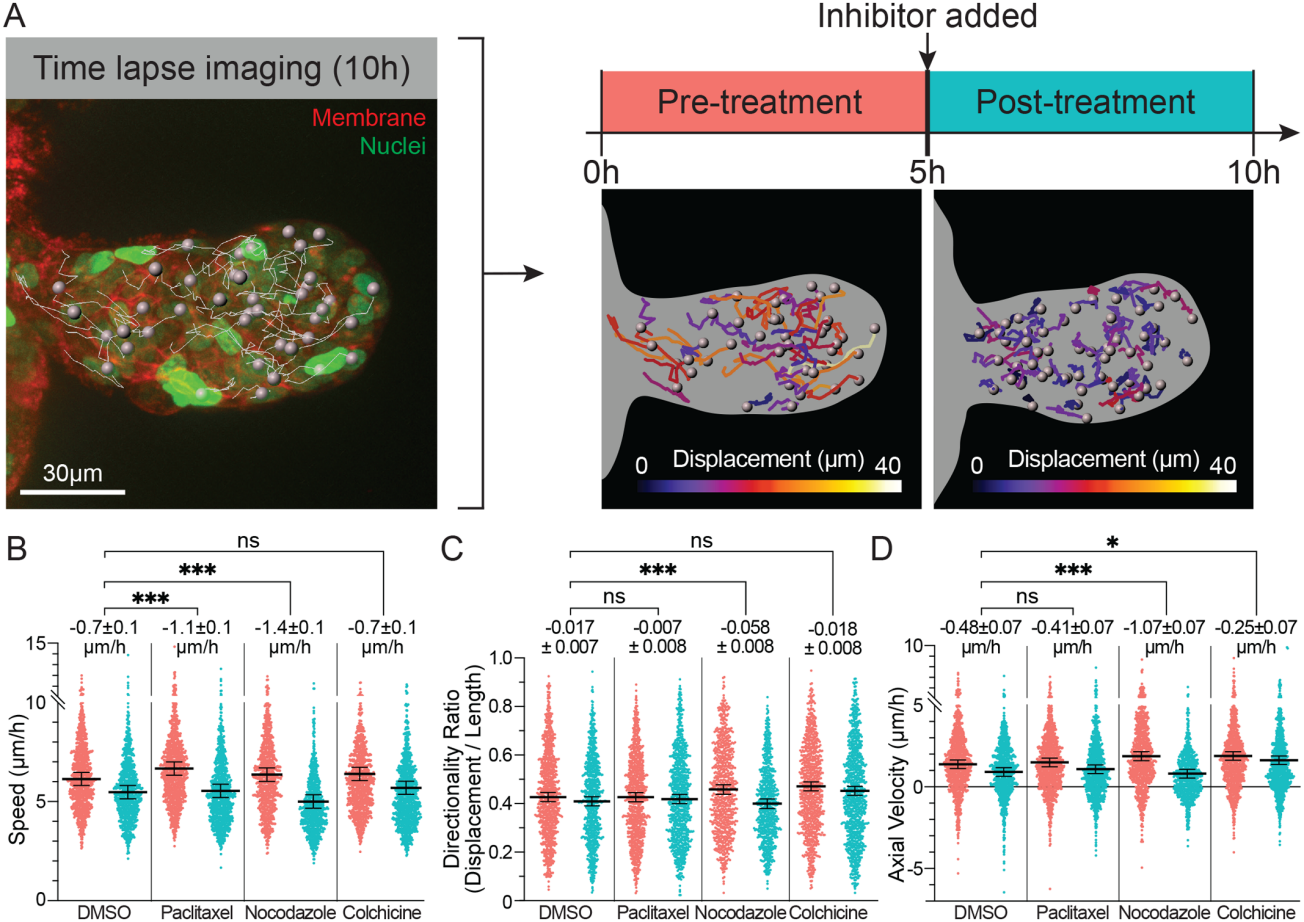
**Microtubule drugs halt TEB elongation by altering the speed and direction of collective migration.** (A) Schematic illustrating cell tracking analysis for a representative bud (red, mTomato; green, H2B-GFP). Quantification of pre-treatment and post-treatment cell migration parameters mean cell speed (B), directionality ratio (C, directionality ratio=track length / net displacement), and axial velocity (D). The positive direction of axial velocity is defined in relation to the line running from the lumen to the front center of the elongating bud. Means±s.e.m. are plotted. All estimated marginal means and statistical significance tests were calculated using a linear mixed effects model. (Each treatment group includes results from r=3 mice and *n*>750 cells from >25 buds).

Mammary epithelial buds elongate primarily through the collective migration of luminal cells (Huebner et al., 2016; Neumann et al., 2018). Since microtubule disruption acutely arrests bud elongation, we hypothesized that microtubules are essential for epithelial cell migration. To test this hypothesis, we used cell tracking analysis to study the effects of microtubule inhibitors on cell motility. Organoids were derived from transgenic mice with fluorescent nuclear and plasma membrane labels (H2B-GFG and mTomato) and cultured until branches formed (days four and five). Using 3D confocal time-lapse, we imaged individual buds for 5 h, before adding inhibitor and imaging the same buds for an additional 5 h. We then followed individual cells within the elongating bud through nuclear tracking, quantifying changes in cell speed and directionality resulting from drug treatment (Fig. 5A). We considered average cell speed as a measurement of cell motility, directionality ratio to assess directional persistence, and axial velocity to assess the extent of migration in the direction of ductal elongation.

We observed a significant effect on all migration metrics post-treatment in the control group due to the intrinsic decrease in bud elongation over time in culture (Fig. 5B-D). Since our primary interest was in determining if responses to inhibitors differed significantly from the response to DMSO, we needed to isolate the treatment response distinct from this general slowing. Mean speed is an average of a cell's instantaneous speed at each recorded timepoint. It measures a cell's overall motility, including both random and directional movement. The reduction in mean speed following paclitaxel and nocodazole treatment was significantly greater than that observed in the vehicle control (Fig. 5B). Both inhibitors decrease motility post-treatment (paclitaxel 17%, nocodazole 22%), but not to a degree that obviously accounts for the magnitude of bud elongation arrest at organoid scale. However, characterization of their effects specifically on directional migration provided clarity. The directionality ratio measures a cell's directional persistence – the fraction of its total movement that contributes to its net displacement. Nocodazole treatment resulted in decreased directionality ratio that was significantly different from control (Fig. 5C). This result suggests that reduced microtubule stability had a more potent effect on directional cell migration than it did on random motility. The reduction in axial velocity was also significant in the nocodazole condition, with a substantial fraction of cells (25.0%) moving in the negative direction back toward the body of the organoid (Fig. 5D). In contrast, paclitaxel treated cells maintained their directionality ratio at least as well as vehicle treated cells (Fig. 5C). Microtubule stabilization therefore appears to preferentially inhibit random motility. Strikingly, the axial velocity changes in paclitaxel treated organoids were not significantly different from that of vehicle (Fig. 5D). Forward directionality was maintained even though cell motility is diminished. Paclitaxel treatment therefore decreases motility, thereby reducing both the radial and random movement, which is important to intercalation in stratified epithelium. The response to colchicine differs from the other inhibitors in that it does not reduce mean speed or reduce the directionality ratio significantly from vehicle control (Fig. 5B,C). Moreover, colchicine treated cells maintained axial velocity post-treatment more effectively than vehicle treated cells (Fig. 5D). Hence, decreased microtubule polymerization biases luminal cell migration in the direction of the tip without decreasing motility overall. This result is consistent with the post-treatment bud elongation observed in colchicine treated organoids. Taken together, our data demonstrate that microtubules are essential to maintaining both the speed and persistence of luminal cell collective migration.

### Induced microtubule severing halts bud elongation

To increase specificity, we also targeted microtubule stability genetically, using a transgenic TRE-spastin mice (Muroyama and Lechler, 2017b). These mice express the microtubule severing protein spastin under the regulation of an inducible (TRE) promotor, as well as CMV-rtTA (Fig. S5A). Administration of doxycycline induces spastin overexpression and rapid disassembly of microtubules. We cultured mammary organoids from these mice until buds began to form on culture day four. We then administered either vehicle or doxycycline to culture media. We detected robust spastin overexpression 24 h after doxycycline treatment, with no detectable expression in vehicle treated controls (Fig. S5B). Buds in vehicle treated organoids continued to robustly elongate and branch (Fig. S5C). Spastin overexpression led to arrest of elongating buds and a significant decrease in organoid growth (Fig. S5C′,D). Small bud-like structures did form after spastin induction, but they failed to elongate and occurred at a significantly reduced rate (Fig. S5C′,D′)*.* These results confirmed genetically that microtubules play an essential role in mammary epithelial bud elongation.

## DISCUSSION

In this study, we set out to understand the role of microtubules in branching morphogenesis. Specifically, we were interested in determining the role of microtubules in mammary luminal epithelial cells during branching morphogenesis. To accomplish this goal, we looked first at the organization, nuclear-centrosomal orientation of microtubules in luminal cells in both simple and stratified epithelium. We found that simple luminal cells utilize apicobasal non-centrosomal arrays and that stratification is accompanied by a switch to radial microtubule organization. The nuclear-centrosomal axis was maintained, with the centrosome remaining at the apical/rear of the nucleus, though transient Golgi reorganization to the leading protrusion was observed during radial intercalation. Second, we looked at the effect of microtubule perturbations on branching morphogenesis. We demonstrated that either stabilization or destabilization of microtubules prevents the initiation of new buds and acutely arrests bud elongation. In contrast, sequestration of tubulin dimers potently inhibited bud initiation but had a modest effect on bud elongation. Finally, we connected microtubule perturbations to effects on collective epithelial migration. Destabilization of microtubules resulted in a loss of cell directionality, preventing coordinated collective migration. Stabilization of microtubules prevented collective migration by reducing luminal cell motility. However, inhibition of tubulin polymerization increased cell migration toward the bud tip.

### Two luminal compartments with distinct microtubule organization

Recent work in the mouse embryonic salivary gland by Wang et al. (2021) contrasts the differences in adhesion, transcription, and behavior between the inner and outer cells of the stratified bud as they drive branching morphogenesis. The differences we observed in cytoskeletal organization suggest a difference in mechanical properties between the boundary layer and inner luminal in mammary buds. In the basally located boundary layer, stable microtubules, cortical tension, and cell-adhesions constrain luminal cells in an epithelial organization. In the stratified layer, dynamic actin, microtubules, and adhesions allow for robust luminal cell rearrangement in an amorphous, fluid-like epithelial collective. Stable intercalation into the boundary layer, and the accompanying cytoskeletal changes, represents a transition between these two phases. What could prompt these changes in the luminal cell and its cytoskeleton? One hypothesis is that stable intercalation is regulated by cell-ECM adhesions formed in the basal-most cell layer. Integrin regulation is essential to mammary epithelial cell migration (Ma et al., 2022), and work in mammary cysts has shown that lumen formation requires sequestration of EB1 microtubule +tips by ILK at the basolateral membrane upon integrin binding at the basal surface (Akhtar and Streuli, 2013). Hence, integrin binding at the tip of an intercalating protrusion could result in boundary capture, microtubule stabilization, and epithelial repolarization.

The difference between luminal compartments is also reflected in the style of migration employed in each compartment. Cells can adapt their migration mode to their mechanical environment – ameboid in soft substrates, mesenchymal in stiff substrates, and hybrid forms in-between (Yamada and Sixt, 2019). Luminal cell migration depends on forward protrusion and rear contractility (Neumann et al., 2018). This result suggests that collectively migrating luminal cells combine elements of ameboid and mesenchymal migration. And indeed, we observed a rear-facing centrosomal MTOC and Golgi within the stratified layer, indicating an ameboid style of movement emphasizing rear tension to drive cells forward (Neumann et al., 2018). However, radial intercalation into the relatively stiffer boundary layer may require a more mesenchymal style, as indicated by the increased protrusive actin assembly and transiently forward-located Golgi. Luminal cells therefore appear to adapt their mode of migration to their physical context within the epithelium. We note that studies utilizing microcontact printed substrates reveal that Golgi positioning is regulated both by migratory mode and by the geometry of the microenvironment through which the cell is migrating (Pouthas et al., 2008). The changing Golgi positioning changing ameboid versus mesenchymal migratory mode, or migration mode and Golgi positioning might independently be responding to the 3D tissue environment.

### Roles of the cytoskeletal systems in epithelial migration

Previous work has demonstrated actin and myosin supply the motive force for collective mammary epithelial migration. Forward-oriented actin protrusions and actomyosin-based rear tension drive radial intercalation within the stratified epithelium, propelling bud elongation and eventually resolving stratified endbuds to simple epithelium (Neumann et al., 2018). Actin and myosin also generate the basal tension maintaining the smooth epithelial boundary (Neumann et al., 2018). Our present study reveals that actin also establishes the mode of migration. Stabilized actin leads to protrusive migration, similar to that observed in stiff collagen gels, while destabilization of cortical structure leads to a loss of collective behavior and integrity of the epithelium-ECM boundary.

While actin dynamics were known to be essential for collective migration, our results demonstrate that microtubule dynamics are also required. Microtubules are essential to directing and supporting actin protrusions, but they must be dynamic to adapt to cell movement. Less stable microtubules were incapable of maintaining directional migration, while more stable microtubules impeded cell migration. Our results are consistent with studies that showed that dynamic microtubules are essential for directed epithelial migration in 2D wound healing assays. Using paclitaxel, nocodazole, and stability altering tubulin mutations, Ganguly et al. (2012) demonstrated that microtubule stabilization inhibited cell migration while destabilization caused a loss in directionality. This concept is consistent with our finding that SIR-Tubulin labeled the non-migratory cells at the basal surface more than the motile cells within the bud. Colchicine also has distinct effects on microtubules, as it sequesters tubulin and limits polymerization, while causing minimal drug-induced microtubule disassembly (Vandecandelaere et al., 1997). Therefore, when polymerization is inhibited, microtubules would still be present to orient migration. This difference could account for the enhanced forward movement observed following colchicine treatment. How colchicine reduces bud branching remains an open question. We speculate that direct effects of 3D cell migration might provide a partial explanation of the benefit of taxane chemotherapies in preventing distant metastatic recurrence in cancer patients.

### Luminal cell states

Taken together, our results suggest a succession of structural states underlying branching morphogenesis. Polarized luminal cells reorganize their apicobasal microtubule arrays to undergo cell division. This change results in low polarity daughter cells with centrosomally organized microtubules. These cells migrate through the stratified layer with their Golgi and centrosome positioned behind the nucleus. This ameboid-like configuration produces interfacial tensions at the rear of the cell that propel migration forward, as shown in (Neumann et al., 2018). As luminal cells intercalate into the outer layer, the Golgi transiently moves anteriorly towards the leading protrusion. At the boundary layer, we observed older, more stable (SiR-Tubulin+) microtubules and an apically localized Golgi. As luminal cells resolve into a simple epithelium behind the bud and undergo apical maturation, microtubules transition from centrosomal to non-centrosomal MTOCs and reform apicobasal arrays. Additional work is needed to characterize the mechanical function and gene expression programs of each luminal cell state and seek to identify the molecular and physical signals that regulate transitions between states. It will also be important for future studies to use higher resolution imaging techniques and labeling approaches that can reveal highly dynamic microtubules, to improve our 4D understanding of microtubule dynamics during branching morphogenesis.

## MATERIALS AND METHODS

### Transgenic animals

A transgenic mouse line expressing tdTomato was used to label cell membranes (Muzumdar et al., 2007) (Jackson Laboratory, #007676). The H2B-GFP mouse line used for cell tracking was a gift from A. K. Hadjantonakis, Memorial Sloan Kettering, USA (Hadjantonakis and Papaioannou, 2004). The transgenic line expressing Centrin-GFP used to identify centrosomes was a gift from S. M. Evans, University of California, San Diego, USA, by way of A. J. Holland, Johns Hopkins University, USA (Hirai et al., 2016). A transgenic line expressing TRE-spastin was a gift from T. Lechler, Duke University Medical Center, USA (Muroyama and Lechler, 2017b). For conditional expression, the TRE-spastin line was crossed with the transgenic CMV::rtTA line gifted by F. Cong and H. Varmus, National Cancer Institute, USA. All other experiments were performed using FVB/NJ strain purchased from Jackson Laboratory. Animal experiments were conducted in accordance with protocols approved by the JHU Medicine Institutional Animal Care and Use Committee.

### Mammary gland organotypic culture

Organoids were prepared and cultured as described in Nguyen-Ngoc et al. (2015). Briefly, mammary glands were dissected from 8-12-week-old female mice and minced with a scalpel. Tissue was treated with trypsin-collagenase and then DNase to digest stromal tissues. Epithelial fragments were recovered through differential centrifugation and resuspended in 4°C ECM solution, equal parts Matrigel (Corning) and pH-neutralized rat tail Collagen I (Corning) at a concentration of 2 organoids/µl. Droplets were plated onto glass-bottom cell culture dishes sitting on a 37°C hot plate. The plated volume per well was adapted to the dish: 50 µl droplet in 96-well plates, 100-150 µl droplet in 24-well plates, 200 µl in four-well chambers, or 10×10 µl droplets arrayed on two-well chambers. Gels were polymerized 30 min in an incubator at 37°C before adding minimal media (DMEM-F12, 1% penicillin-streptomycin, 1% insulin-selenium-transferrin) or branching media (minimal media, 2.5 nM FGF2). Cultures were incubated at 37°C and 5% CO_2_ for up to 8 days.

### Differential interference contrast (DIC) microscopy

Images and timelapse movies of organoid cultures were recorded on a Zeiss Cell Observer with a 20× objective, an AxioObserver Z1 equipped for DIC imaging, and an AxioCam MRM camera. During timelapse, up to 400 organoids were imaged in parallel every 20 min for 2-5 days. Image acquisition was briefly paused for media changes, when necessary. Organoids were maintained at 37°C and 5% CO_2_ throughout the experiment. For a more detailed methodology, please see Ewald (2013).

### Super-resolution live imaging of sir-tubulin

Organoids were cultured in 200 µl gels in four-well chambers. Organoids were cultured until active branching was evident, typically at day five of culture, then prepared for imaging. Two hours before imaging, Sir-Tubulin as added to organoid culture media (50 nM, 1:1000 DMSO); at this dose it is not expected to induce changes in microtubule dynamics. Cultures were maintained at 37°C and 5% CO_2_ during imaging on a Zeiss LSM880 equipped with Airyscan FAST super-resolution module. Image stacks (220 nm Z-step) were acquired in Airyscan FAST mode using a 40×/1.4 C-Apo water immersion objective and Zen Black acquisition software. Image files underwent Airyscan Processing in Zen Black before Z-projection (five slices, 1 µm). Brightness and contrast adjustments were made in FIJI, across the whole image, to maximize clarity.

### SirTubulin intensity quantification

Differences in microtubule staining intensity was quantified from live imaging of SirTubulin in actively elongating TEBs. Imaging was typically done around day five of culture. Three representative areas were selected on the outer layer of cells at the leading edge of elongation and three corresponding areas were selected from the inner of cells. See Fig. S1A for an example of sample region pairs. ImageJ was used to measure the average intensity of both SirTubulin and mTomato signal in these areas. Analysis was performed across six TEB units from two separate biological replicates. GraphPad Prism was used to plot the average signal intensity of each inner-outer cell pair and quantify the statistical significance of the difference in intensity between inner and outer cells.

### Fixation and immunofluorescence staining

Organoids were cultured in 10 µl gels arrayed in two-well chambers. Media was removed and wells rinsed with 37°C D-PBS. The chambers were filled with −20°C methanol and immediately placed on a block prechilled to −20°C and placed in −20°C freezer for 20 min. Samples were rinsed with TBS before individual gels were transferred with forceps into a 48-well plate. They were then permeabilized (20 min, TBS+0.5% Triton X-100), and blocked in IF buffer+5% BSA (45 min, TBS+0.05% Tween20+0.2% Triton X-100) at room temperature. Immunofluorescence was performed by incubating with primary antibodies for 1 h at room temperature or overnight at 4°C. Samples were then washed 2×15 min, incubated with secondary antibodies and DAPI (1:10,000) for 1 h at room temperature, and washed 3x in PBS. See Table 1 for a list of antibodies used. Stained gels were transferred with forceps into a glass bottom 96*-*well plate and incubated for 30 min in antifade mounting medium before imaging. Airyscan imaging and processing was performed as described above, except that a z-step of 0.5 µm was used when microtubules were not being imaged.

**Table 1.** Antibodies used in immunofluorescence experiments

Antibody	Species	Dilution	Source
α CAMSAP3	Rabbit	1:200	Gift from M. Takeichi (Tanaka et al., 2012)
α Zo-1	Rat	1:100	Santa Cruz Biotechnology, sc-33725
α GM130	Mouse	1:200	BD Biosciences, 610822
α alpha-tubulin	Rat	1:200	Millipore, T9028
α tubulin (polyclonal)	Sheep	1:200	Cytoskeleton Inc., ATN02
α HA-tag	Mouse	1:1000	Thermo Fisher, 26183
α Cleaved Caspase3	Rabbit	1:200	Cell Signaling Technology, 9661
α-Rb Alexafluor-405	Goat	1:200	Thermo Fisher, A-31556
α-Rb Alexafluor-488	Goat	1:200	Thermo Fisher, A27034
α-Rt Alexafluor-488	Goat	1:200	Thermo Fisher, A-11006
α-Sheep Alexaflour-488	Donkey	1:500	Jackson Immunoresearch, 713-545-003
α-Rt Alexafluor-647	Goat	1:200	Thermo Fisher, A-21247
α-Ms Alexafluor-647	Goat	1:200	Thermo Fisher, A28181

### Spinning disk confocal live imaging of bud elongation

Organoids were cultured in 150 µl gels in 24-well plates. Cultures were maintained at 37°C and 5% CO_2_ during imaging on a spinning-disk confocal microscope (Solamere Technology Group Inc.). Images were acquired using a 40×/1.2 C-Apo water immersion objective with Immersol and MicroManager acquisition software. Up to 200 positions of interest were selected and recorded in each experiment. Image stacks (17×2 µm) were collected at each position every 20 min for up to 16 h. Images were saved as TIFF-image stack files and visualized using Imaris 9 adjusting brightness and contrast to maximize image clarity.

### Flow cytometry

Organoids were prepared for flow cytometry as described in Padmanaban et al. (2020). Briefly, organoids were recovered from gels by collagenase digestion followed by a wash with ice cold EDTA recovery solution and then trypsinized to single cells. Each experiment was repeated in at least three independent biological replicates. Cell proliferation was accessed using Click-It EDU (Thermo Fisher) kit. Culture media was supplemented with EdU 24 h in advance of collection and digestion. Cells were fixed, permeabilized, and conjugated with Alexafluor488 according to the manufacturer's directions. Cell cycle was accessed using FXCycle-PI (Thermo Fisher). Cells were fixed, washed, and stained according to the manufacturer's directions. Flow experiments were performed on a Thermo Fisher Attune Flow Cytometer, gating for single luminal cells based on forward scatter and side scatter. Analysis was performed in FlowJo, specifically using the cell-cycle curve fitting tool to determine cell-phase distribution. Within each biological replicate, all measurements were normalized to the vehicle condition to control for intrinsic variation between replicates. Graphing and statistical analysis was performed in Graphpad Prism. The statistical significance of treatment response was determined using the non-parametric Wilcoxon signed-rank test (vehicle median=1, *P*=0.05).

### Drug screening

For drug screening experiments, organoids were prepared and suspended in ECM gel solution as described above. Gels were plated using a Thermo Fisher liquid handling system. The liquid handling reservoir was prechilled in 4°C deli fridge and then packed in ice during the plating process. Gel solution was aliquoted into 24- or 96-well plates on a 37°C heating pad. Organoids were incubated overnight in minimal media before changing to branching media. Drug treatments were prepared at 2× concentration using a Tecan D300e Digital Dispenser and then added in equal volume to media in organoid culture wells to obtain desired dosing. In branching initiation experiments, inhibitors were introduced upon addition of branching media (day one). In branching elongation experiments, drugs were added once most organoids had formed buds (∼day four). On culture day seven, culture media was supplemented with Ethidium Homodimer (EtHD, 3 µM) and incubated for 20 min before imaging. Plates were imaged live using a Molecular Devices High-Content Imaging system. Tiled image stacks covering each well were collected in brightfield and EtHD channels (15×100 µm z-step, 4× objective). Viability was accessed from maximum intensity z-projections by measuring the average intensity of EtHD fluorescence within the organoid boundaries, which were identified from brightfield images using the automated boundary detection FIJI-macro (Hasnain et al., 2020). The statistical significance of differences between treatment conditions and aphidicolin control were determined using a nested one-way ANOVA with Dunnett's correction for multiple comparison (*P*=0.05). In bud initiation assays, branching was accessed by quantifying the percentage of organoids with three or more buds (% branching), and variable-slope inhibition dose response curves were fit using non-linear regression in GraphPad Prism. In bud elongation assays, organoid boundaries were traced manually in FIJI to measure organoid size (area of traced shape) and branching (bud count). The statistical significance of treatment response was determined using the non-parametric Wilcoxon signed-rank test (vehicle median=1, *P*=0.05). Within each biological replicate, measurements were normalized to the corresponding vehicle condition to control for intrinsic variation in initial organoid size. All graphing and statistical analysis was performed in GraphPad Prism. All results represent a minimum of three biological replicates.

### Nuclear tracking and cell migration analysis

Spinning disk confocal imaging was performed as described above, except that image acquisition was paused after 6-7 h to add inhibitors to the media and then adjust the focus and stage positioning before resuming image acquisition. Four independent replicates were performed in this way, recording up to 150 bud positions in each experiment.

Images were prepared for cell tracking analysis by performing background subtraction in FIJI, (rolling ball=50). Image sequences showing actively elongating buds were selected for analysis and imported to Imaris 9. A gaussian blur (1 pixel) and thresholding was applied to each image in Imaris batch, to aid automated spot detection. Shifts in sample position occurring during media changes were corrected by manual frame of reference adjustment. Sample drift was corrected by performing a preliminary spot tracking in batch and then individually applying the “Correct Drift” function to cells tracks in the stalk region in each image. A computational reference frame was defined for each bud with y-axis oriented in the direction of collective migration and origin centered at the rear-boundary of the stratified region. Trajectories were determined using a final batch spot tracking (Spot: Radius=4.5 µm, Quality>5; Track: Method=Autoregressive Motion, max timepoint displacement=7 µm, max gap between spots=3). Spots outside the bud structure or corresponding to cells partially obscured by image boundaries or were manually removed. Track and spot statistics were exported as CSV files.

Data cleaning and computation was performed in R using the tidyverse package. First, cell data from the 5 h immediately before and after inhibitor addition was extracted from the full dataset and separated into pre-treatment and post-treatment groups. Then, the mean speed, directionality ratio, and axial velocity was calculated for every cell that originated in the stratified region and was tracked for at least 3 h. Preliminary analysis flagged buds that were significant outliers in at least one migration metric. These outliers were reviewed in Imaris to correct any sample drift missed on the first pass. The corrected tracking data was exported and analyzed once again. Final analysis considered elongating buds (average pre-treatment axial velocity >0) with ten or more cell tracks. See Table 2 for a summary of the number of buds and cells tracked from each independent replicate.

**Table 2.** Number of cells tracked per condition and replicate

		# of buds	# of cells tracked	
	Experiment	Analyzed	Pre-treatment	Post-treatment
Colchicine	1	8	241	274
	2	13	436	512
	3	4	59	49
	4	6	149	126
	Total	31	885	961
Nocodazole	1	6	290	220
	2	10	363	387
	3	6	101	104
	4	4	67	50
	Total	26	821	761
Paclitaxel	1	5	187	223
	2	9	291	286
	3	12	282	233
	4	5	93	89
	Total	31	853	831
Vehicle	1	8	420	376
	2	4	130	139
	3	7	188	185
	4	10	203	151
	Total	29	941	851

Statistical analysis was performed in R using the stats, lme4, and lmerTest packages. Cell tracking data was analyzed using linear mixed effects models to account for repeated measures and inherent biological variability of the experimental and biological replicates. The model included the fixed effects of treatment status (Pre-Treatment, Post-Treatment), experimental condition (DMSO, Paclitaxel, Nocodazole, and Colchicine), and the interaction of the two factors. The model also included random intercepts for bud and experiment replicates. The dependent variable was one of the values of interest – Average Speed, Directionality Ratio, or Axial velocity. The model was used to determine pre-treatment and post-treatment least squares means and the mean treatment response for each inhibitor condition as well as to compare the mean treatment response for each inhibitor to that of DMSO control. If multiple inhibitors showed a statistically significant difference (*P*<0.05), the treatment responses of those inhibitors were compared by simultaneous general linear hypothesis testing. Graphs were prepared in Graphpad Prism.

### Induction of spastin expression

Organoids from TRE-Spastin mice were prepared as described and cultured for 4 days before DIC timelapse imaging. Once bud initiation occurred, media was supplemented with vehicle (PBS) or doxycycline (30 µM). Doxycycline enabled rtTA activation of Tet-on promotor to induce Spastin expression. Timelapse imaging continued for an additional 24 h, at which point organoids were fixed in PFA and immunolabeled (Neumann et al., 2018) for cleaved-caspase3 and Spastin HA tag (Table 1). Fluorescent images were taken using the spinning disk confocal microscope described above and visualized in FIJI, adjusting brightness and contrast, across the whole image, to improve clarity. In the timelapse movies, projected area and bud number was quantified at the initial and final timepoint for each organoid by boundary tracing in FIJI. Growth and branching were accessed by calculating the ratio between final and initial area and bud number for each organoid. Results reflect results from two biological replicates in independent experiments.

## Supplementary Material

10.1242/biolopen.062267_sup1 Supplementary information
