# Supplementary material for: Microtubule dependent changes in cell polarity are required for mammary epithelial cell migration and branching morphogenesis
Source: Biol Open. 2025 Dec 11;14(12):bio062267. doi: 10.1242/bio.062267 (PMC12746711; doi:10.1242/bio.062267)
Supplement: Supplementary information [file biolopen-14-062267-s1.pdf]

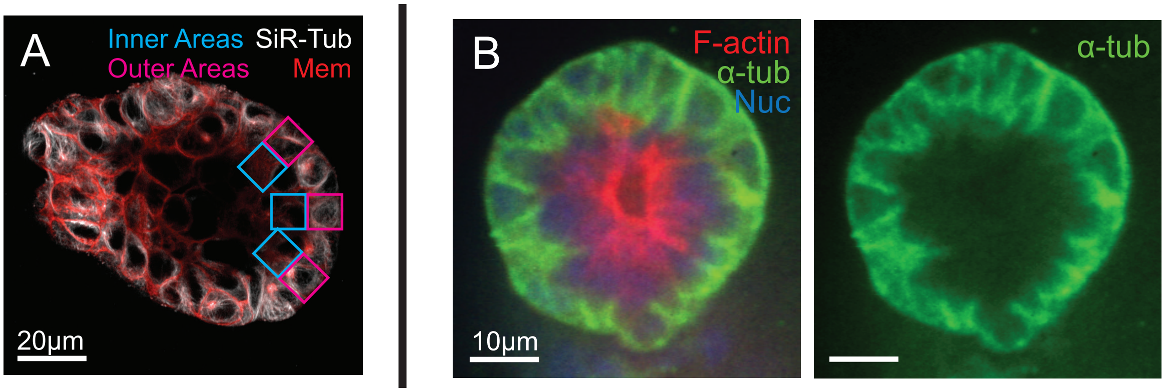

Fig. S1. MTOC organization and microtubule stability during branching morphogenesis. (A) Schematic showing example of fluorescent intensity sampling of three locations of parried inner and outer stratified layers. (B) Confocal IF imaging of microtubules in stratified mammary epithelium (red = F-actin, green = α-tubulin, blue = DAPI).

Biology Open • Supplementary information

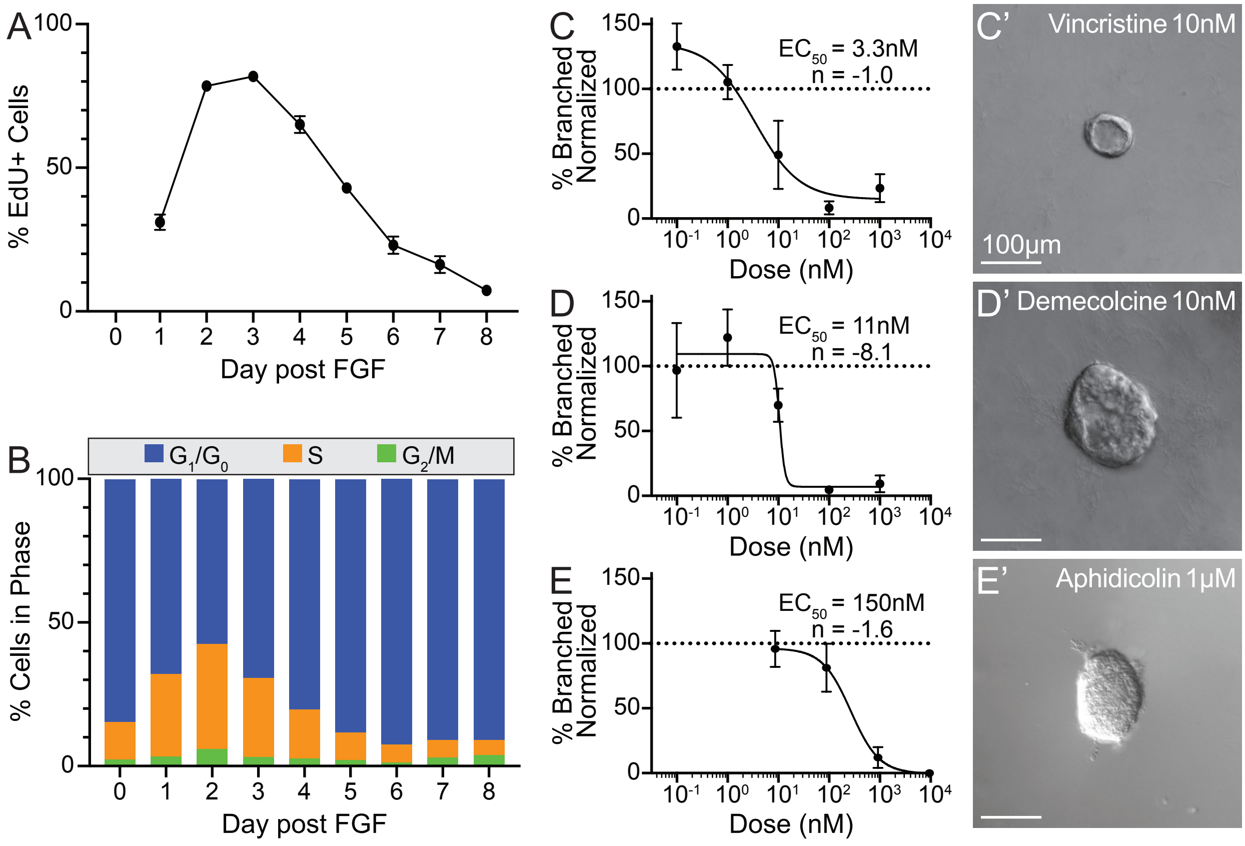

Fig. S2. Proliferation dynamics in mammary organoids following treatment with FGF2. (A) FACS-based analysis of proliferation rate of mammary epithelial cells in organoid culture as a function of time. Proliferation is accessed by percentage of cells that incorporated EdU in the previous 24 hours (r = 3). (B) FACS-based cell cycle profile of mammary epithelial cells isolated from a series of timed organoid cultures. Cell cycle was determined by DNA content (FxCycle PI, r = 3 mice). (C-C') Dose response and representative day 7 DIC images for microtubule destabilizer vincristine, (D-D') microtubule polymerization inhibitor demecolcine, and (E-E') DNA synthesis inhibitor aphidicolin. (r = 3 mice, for each condition).

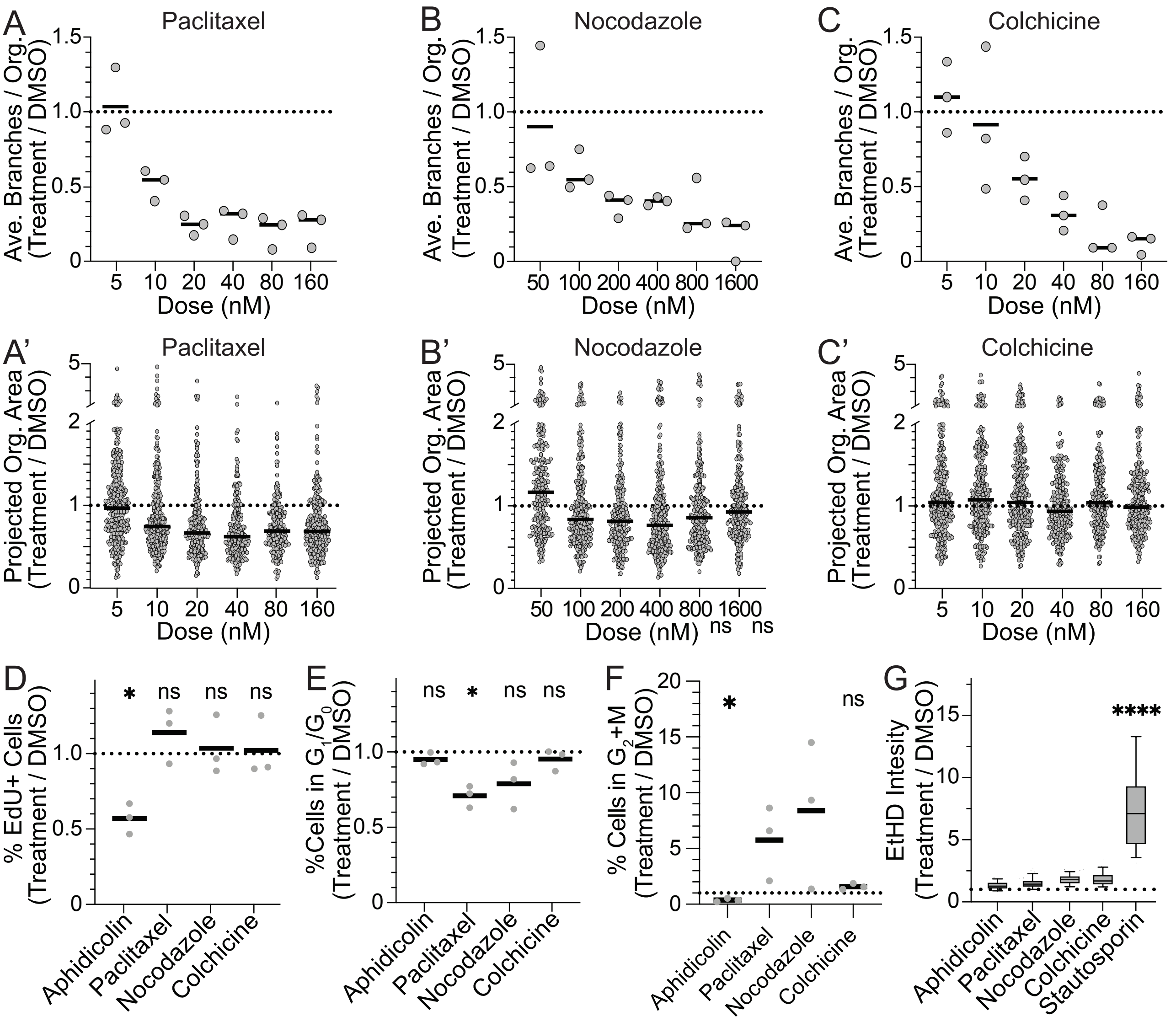

Fig. S3. Dose response curves for microtubule inhibitors during branching stage. Drugs were added to culture media after bud initiation and accessed 2 days later (r = 3 mice, n >= 200 orgs.). (A-C) Quantification of MTA effects on branch formation (average[buds per organoid]$_{treatment}$ / average[buds per organoid]$_{DMSO}$) and (A'-C') organoid growth (projected area$_{treatment}$ / projected area$_{DMSO}$). Effects of MTA addition to actively elongating organoids on cell EdU incorporation (D), percentage of cells in G_1/G_0 (E), percentage of cells in G2+M (F), and cell death (G).

Biology Open • Supplementary information

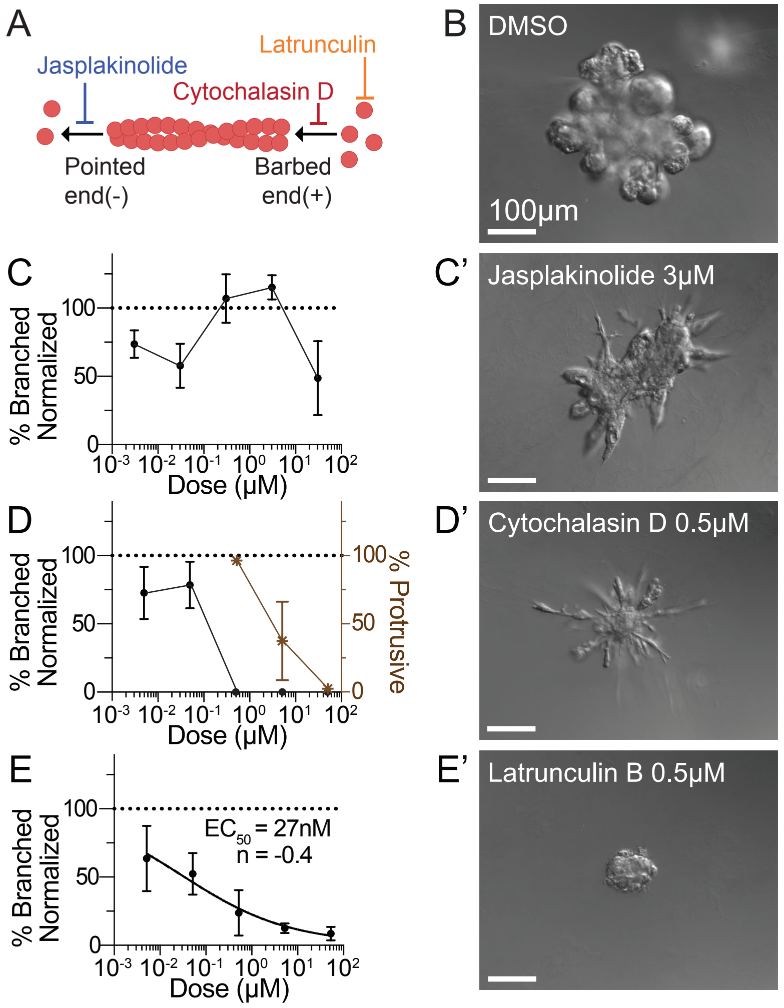

Fig. S4. Altered actin dynamics change the mode of epithelial migration. (A) Schematic illustrating the mechanism of action for actin inhibitors jasplakinolide (C-C' stabilizes F-actin), cytochalasin D (D-D', destabilizes F-actin), and latrunculin B (E-E', binds free G-actin). (C-E) Branching response as a function of drug concentration (r = 3 mice). (B,C'-E') Representative DIC images from DMSO control and treatment groups.

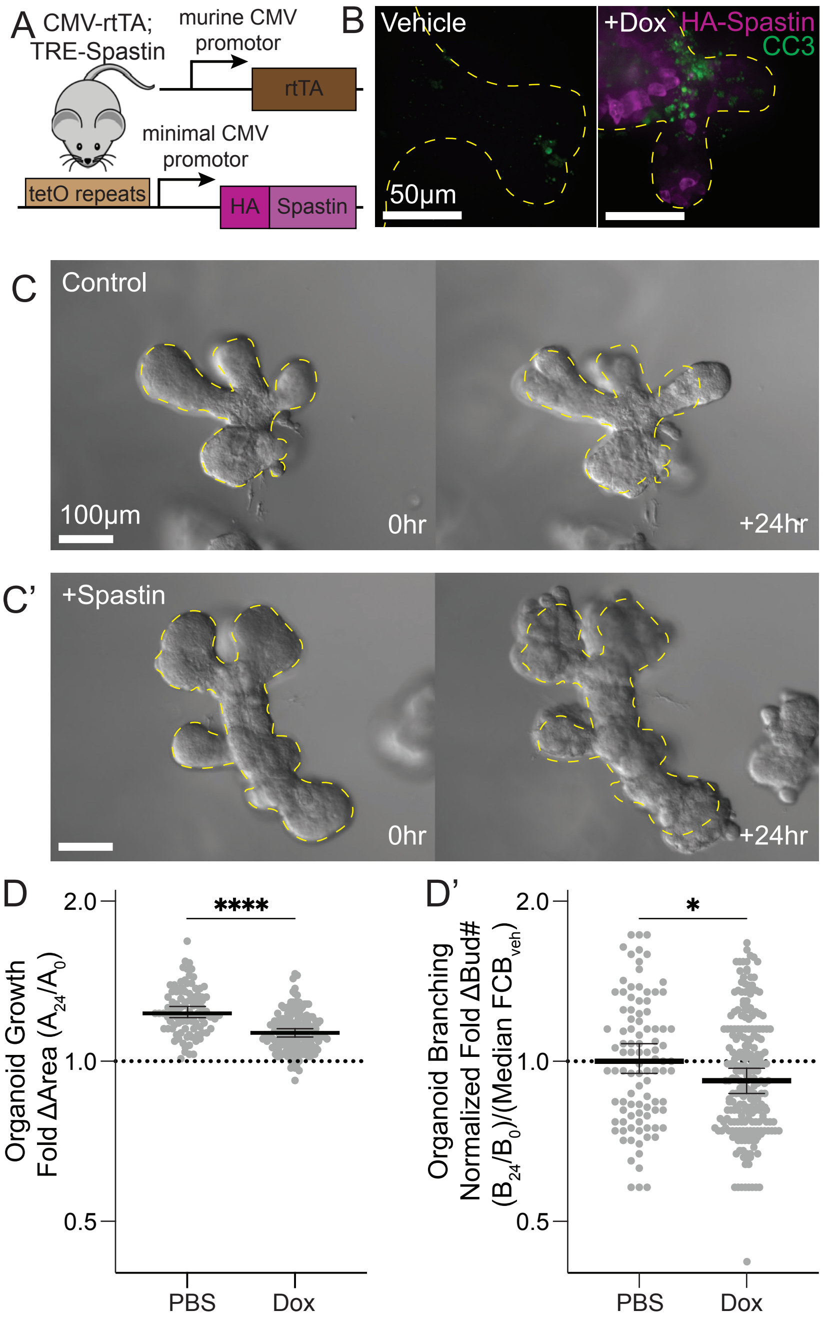

Fig. S5. Induced microtubule severing halts bud elongation. (A) Schematic illustrating the genetics of TRE-spastin mice (mouseCMV :: rtTA, tet-On/minimalCMV :: spastin-HAtag). (B) Representative IF images depicting spastin-HA (magenta) and cleaved-Caspase3 (CC3, green) in mammary buds following 24 hrs vehicle or doxycycline treatment. (C-C') Representative DIC images of mammary organoids before and 24 hrs after addition of vehicle or doxycycline (treatment at day 6). Quantification of organoid growth (D) and branching (D') during 24hr treatment period. (r = 3 mice, n > 100 organoids).

Biology Open • Supplementary information

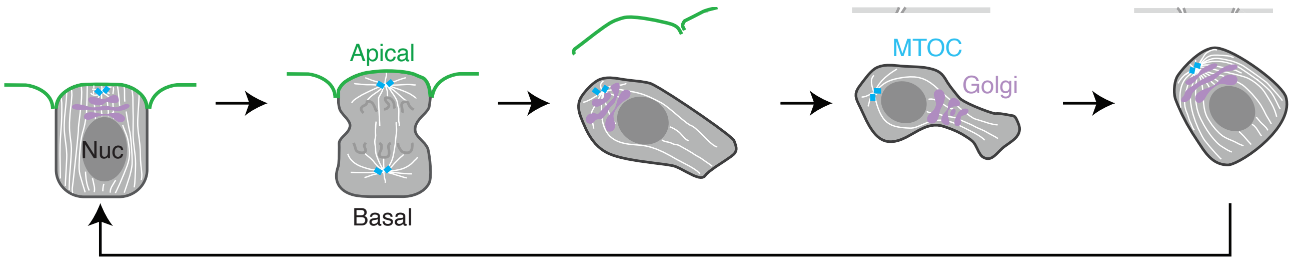

Graphical Abstract: Proposed model for luminal cell microtubule organization states. Luminal cells reorganize their microtubules (MTs) as they progress through stratification, collective migration, radial intercalation, and repolarization during branching morphogenesis. The nucleus (Nuc), the apical and basal surfaces of the cell, the centrosomal MTOC and the Golgi are labeled.

Table S1. Antibodies used in immunofluorescence experiments

Antibody	Species	Dilution	Source
α CAMSAP3	Rabbit	1:200	Gift from M. Takeichi (Tanaka et al., 2012)
α Zo-1	Rat	1:100	Santa Cruz Biotechnology, sc-33725
α GM130	Mouse	1:200	BD Biosciences, 610822
α alpha-tubulin	Rat	1:200	Millipore, T9028
α tubulin (polyclonal)	Sheep	1:200	Cytoskeleton Inc., ATN02
α HA-tag	Mouse	1:1000	Thermo Fisher, 26183
α Cleaved Caspase3	Rabbit	1:200	Cell Signaling Technology, 9661
α-Rb Alexafluor-405	Goat	1:200	Thermo Fisher, A-31556
α-Rb Alexafluor-488	Goat	1:200	Thermo Fisher, A27034
α-Rt Alexafluor-488	Goat	1:200	Thermo Fisher, A-11006
α-Sheep Alexaflour-488	Donkey	1:500	Jackson Immunoresearch, 713-545-003
α-Rt Alexafluor-647	Goat	1:200	Thermo Fisher, A-21247
α-Ms Alexafluor-647	Goat	1:200	Thermo Fisher, A28181

Biology Open • Supplementary information

Table S2. Number of cells tracked per condition and replicate

		# of Buds	# of Cells Tracked	
	Experiment	Analyzed	Pre-Treatment	Post -Treatment
Colchicine	1	8	241	274
	2	13	436	512
	3	4	59	49
	4	6	149	126
	**Total**	**31**	**885**	**961**
Nocodazole	1	6	290	220
	2	10	363	387
	3	6	101	104
	4	4	67	50
	**Total**	**26**	**821**	**761**
Paclitaxel	1	5	187	223
	2	9	291	286
	3	12	282	233
	4	5	93	89
	**Total**	**31**	**853**	**831**
Vehicle	1	8	420	376
	2	4	130	139
	3	7	188	185
	4	10	203	151
	**Total**	**29**	**941**	**851**

Biology Open • Supplementary information
